# Geographically Sourcing Cocaine’s Origin – Delineation of the Nineteen Major Coca Growing Regions in South America

**DOI:** 10.1038/srep23520

**Published:** 2016-03-23

**Authors:** Jennifer R. Mallette, John F. Casale, James Jordan, David R. Morello, Paul M. Beyer

**Affiliations:** 1 U.S. Drug Enforcement Administration, Special Testing and Research Laboratory, Dulles, VA 20166 USA; 2 National Geospatial Intelligence Agency, Springfield, VA 20150 USA; 3 U.S. Drug Enforcement Administration, Intelligence Division, Arlington, VA 22202 USA

## Abstract

Previously, geo-sourcing to five major coca growing regions within South America was accomplished. However, the expansion of coca cultivation throughout South America made sub-regional origin determinations increasingly difficult. The former methodology was recently enhanced with additional stable isotope analyses (^2^H and ^18^O) to fully characterize cocaine due to the varying environmental conditions in which the coca was grown. An improved data analysis method was implemented with the combination of machine learning and multivariate statistical analysis methods to provide further partitioning between growing regions. Here, we show how the combination of trace cocaine alkaloids, stable isotopes, and multivariate statistical analyses can be used to classify illicit cocaine as originating from one of 19 growing regions within South America. The data obtained through this approach can be used to describe current coca cultivation and production trends, highlight trafficking routes, as well as identify new coca growing regions.

Cocaine remains one of the most widely used narcotics in the world, and the United States is a primary consumer^1^. The widespread abuse of cocaine has led to numerous investigations into its production and characterization^2^,^3^,^4^,^5^,^6^. In the past, profiling studies have focused on the isolation of trace alkaloids present in illicit cocaine with the intent of comparing and thus linking samples seized by law enforcement agencies^7^,^8^,^9^,^10^. Comparison analyses provide valuable information; however, due to the movement of cocaine for processing and distribution between multiple locations, successful sample-to-sample association is often difficult. Chemical profiling does, however, have significant merit in building the foundation for determining the origin of illicit cocaine. Geographically sourcing cocaine not only addresses the complex movement of cocaine but may also influence law enforcement’s coca eradication and cocaine interdiction strategies.

Cocaine origin determinations were first successfully accomplished through the utilization of trace alkaloid data in combination with stable isotope ratios (δ) of purified cocaine^11^. Five coca growing regions throughout South America were investigated: The Chapare Valley in Bolivia, the Huallaga/Ucayali and Apurimac Valleys in Peru, and the Guaviare and Putumayo-Caquetá regions in Colombia. Five variables were considered: tropacocaine, trimethoxycocaine, total truxilline content, and two stable isotope ratios, δ^13^C and δ^15^N. The alkaloid content of cocaine is primarily indicative of the coca variety utilized for production, as the minor plant alkaloids that are carried through illicit processing vary by cultivar. Additionally, the prevalence of each variety differs throughout South America, thus indicating a probable region of growth^3^,^4^,^5^. Recent analyses of coca varieties and numerous cultigens have further enhanced our understanding of the known variations of alkaloid content within cocaine^12^. Stable isotope ratios are utilized in various fields of study for geographical origin determinations and predicting environmental patterns^13^,^14^,^15^. The stable isotopes present in cocaine are unaffected by illicit processing methods^16^ and are therefore a reflection of the environment in which the coca was cultivated.

Due to the natural variations in alkaloids and stable isotopes, characteristic profiles were easily identified and thus provided the basis for classification of cocaine by country of origin, i.e., Bolivia, Colombia, or Peru. Country-of-origin classifications were highly accurate (>95%), and regional differences were noted. Over the past 15 years, however, the data have exhibited less definitive separation between the classic regions due to the expansion of coca cultivation. In the early 2000s, cultivation in Colombia began to increase exponentially in response to law enforcement efforts to eliminate the shipment of cocaine base from Peru. The five aforementioned regions are now only part of a list of 19 known coca growing regions (Fig. 1). Despite the large shift in coca cultivation, assignment of cocaine to its source country is still possible with the previous methodology, but resolution between specific regions has been greatly reduced.

In order to improve the discriminatory ability of the existing geo-sourcing analyses, two measurements were added to the analysis scheme: δ^2^H and δ^18^O. Hydrogen and oxygen isotopic differences are expected in cocaine throughout South America due to the effects of precipitation and humidity conditions on isotope incorporation in plants^17^,^18^. Until now, however, this type of data has been unexplored for cocaine. Furthermore, an updated data analysis scheme based on exploratory spatial data analysis (ESDA) and chemometrics based multivariate statistical analysis methods was developed to simultaneously evaluate all collected data and visualize trends among the known growing regions under study. This provided the foundation for assigning accurate sub-regional classifications to illicit cocaine samples. By incorporating additional stable isotope analyses and multivariate statistical analyses to the existing geo-sourcing program, we have successfully developed methodology to classify illicit cocaine as originating from one of 19 known coca growing regions within Bolivia, Colombia, and Peru.

## Results

**Figure 1:**
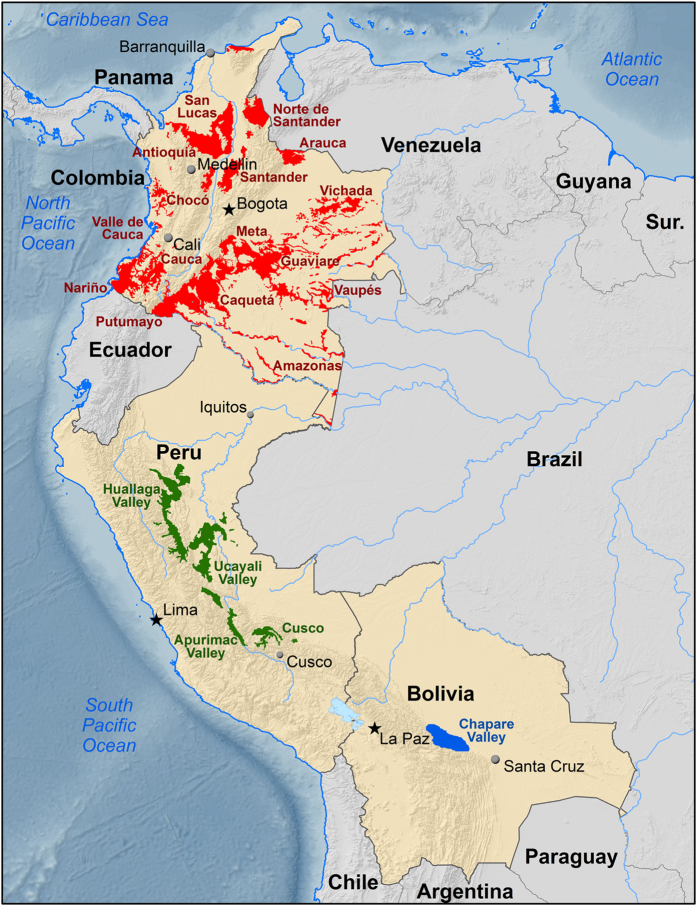
Map outlining each of the known South American coca-growing regions investigated. (Colombia = red; Peru = green; Bolivia = blue). The map was created with ArcGIS Advanced software (Environmental Systems Research Institute).

More than 500 samples of coca leaf from the following coca-growing regions were analyzed: the Chapare region of Bolivia; the Amazonas, Antioquia, Arauca, Caquetá, Cauca, Chocó, Guaviare, Meta, Nariño, Norte de Santander, Putumayo, San Lucas, Santander, Valle de Cauca, Vaupés, and Vichada regions of Colombia; and the Cusco/Apurimac (Cusco) and Ucayali/Huallaga Valleys (UHV) of Peru. All regions are illustrated in Fig. 1. Cocaine was extracted, purified, and analyzed for total alkaloid content. Additionally, the isotopes δ^13^C, δ^15^N, δ^2^H, and δ^18^O were accurately measured in every sample. Average results from the analyses are listed in Table 1.

**Figure 2:**
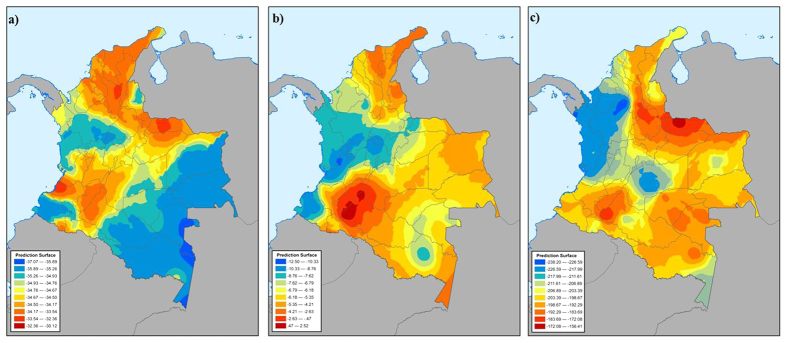
Cocaine isoscapes of (**a**) δ^13^C, (**b**) δ^15^N and (**c**) δ^2^H in Colombia interpolated from 336 authentic coca leaf samples. All displayed values are in the traditional isotopic unit, ‰. The isoscapes were created with ArcGIS Advanced software (Environmental Systems Research Institute).

**Figure 3:**
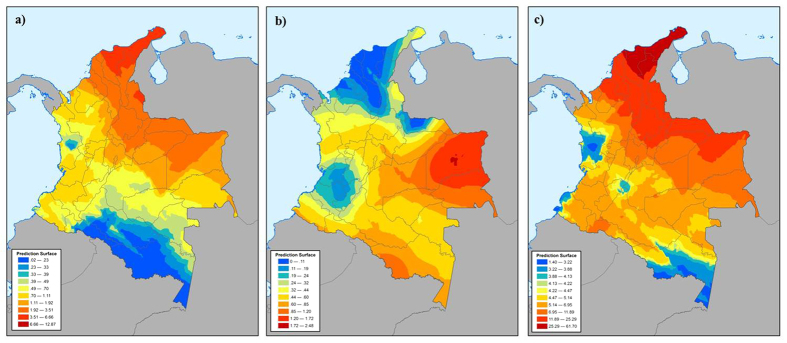
Cocaine alkaloid landscapes (alkaloidscapes) of (**a**) tropacocaine, (**b**) trimethoxycocaine, and (**c**) total truxillines in Colombia interpolated from 336 authentic coca leaf samples. All values are displayed at % relative to cocaine. The data landscapes were created with ArcGIS Advanced software (Environmental Systems Research Institute).

Each collected coca leaf sample was referenced with the geographical coordinates of the originating field in addition to the general sub-regional location. The geospatial nature of the data provided a means to visualize each variable across a very large area. As illustrated in Fig. 2, the isotopes measured in the cocaine from Colombia were utilized to model the expected cocaine isotope measurements throughout the entire country. This type of data representation is referred to as an “isoscape,” and is a fundamental method to illustrate the expected results of a particular measurement based on actual observations from a smaller sample set^19^,^20^. In this study, the kriging interpolation method^21^ was utilized to produce each isoscape, and parameters were selected in order to produce the least amount of error within the landscapes. The same treatment was applied to the alkaloid data as shown in Fig. 3. The ordinary kriging method was selected with the intent to illustrate the variation of each measurement only in terms of location. This process is not part of the final determination of origin for cocaine samples, but is instead used for a visualization aid when identifying and understanding general differences observed throughout Colombia.

### Alkaloids (Tropacocaine, Trimethoxycocaine and Truxillines)

Figures 2 and 3 illustrate the general trend of each analytical response for cocaine within Colombia. Tropacocaine, for example, is present in higher amounts (>1%) in samples originating from northeastern Colombia (Arauca, San Lucas, Norte Santander, etc.). Cocaine from southwestern Colombia typically has lower amounts of tropacocaine (Nariño, Cauca, Putumayo, etc.). The total alkaloid content in cocaine from Peru and Bolivia has been well established^11^, and the tropacocaine present within these samples is relatively low, <0.25% (Table 1). Similarly, trimethoxycocaine is generally lower in samples from Peru and Bolivia than those from Colombia. However, samples originating from southwestern Colombia generally have similar trimethoxycocaine values to Peruvian and Bolivian cocaine, thus making a distinction between the three source countries difficult. Total truxilline content is useful since most Colombian samples contain more than 5%. However, there are regions within Colombia that contain cocaine with lower truxilline content (Nariño, Chocó, etc.). This type of overlap further complicates sourcing any sample to a sub-region and possibly country if only trace alkaloid data is considered.

### Stable Isotopes (δ^13^C, δ^15^N, δ^2^H and δ^18^O)

The isotopic trends displayed in all four stable isotopes are consistent with predicted patterns for plants (Fig. 2). Fundamental carbon isotope theory describes how predictable patterns are often observed and are typically based on environmental changes^22^,^23^,^24^. Traditional theories hold true for cocaine as well, since the environmental effects on the coca plant are reflected by the isotopic signature observed in cocaine. The δ^13^C pattern of cocaine is more enriched along the regions of Caquetá and Cauca and through the center of Colombia, up to the northeastern border. As expected, this trend loosely follows along the Andean mountain range including the regions of Santander, Antioquia, and San Lucas. The areas of depleted δ^13^C correspond to regions of lower elevation found in regions such as Amazonas, Chocó, and Vichada. These effects are directly related to temperature and CO_2_ differences along altitude gradients due to the subsequent influence on stomate behavior in plants^25^,^26^.

In general, δ^15^N is often more enriched in samples originating from Colombia in comparison to those from Bolivia or Peru. This observation is pronounced in the southern regions of Putumayo and Caquetá. Upon moving further south into Peru, δ^15^N is depleted, and this pattern is continued into Bolivia, with data similar to that observed in the Colombian regions of Chocó, Santander, and Valle de Cauca. The levels of δ^15^N present in cocaine throughout South America are consistent with the known effects of precipitation and soil type on nitrogen cycling patterns in comparison to plants grown in temperate climates. In tropical environments, ^15^N enrichment suggests increased nitrogen availability in the soil and plant; however, there may not always be a direct correlation because nitrogen cycling rates and patterns are also affected by soil and plant types within a specific region^27^.

Deuterium and oxygen isotope ratios in plants and plant products have been shown to be directly influenced by humidity conditions and source water^17^,^18^. Deuterium and oxygen isotope ratios in cocaine exhibit these effects as well. Since deuterium and oxygen are so closely related and often reflect the same patterns, only the isoscape for deuterium is shown in Fig. 2. The pattern illustrated reflects the depletion of deuterium in central Colombia starting from the western coast and continuing across the Andean mountain range (Antioquia, Meta, Chocó, etc). This observation is expected and explained by the change in humidity and precipitation patterns across elevation. As cloud systems move further inland, precipitation events become more depleted. The southwestern regions of Nariño and Cauca don’t necessarily follow this trend, and the enrichment may be due to wind and weather patterns originating from Ecuador rather than directly off of the Pacific Ocean. It is also noted deuterium is enriched moving inland along the lower lying valleys and plains of Colombia (Arauca, Norte de Santander) likely due to the multiple evaporation/precipitation events occurring. Overall, the deuterium/oxygen isotope patterns in Peru and Bolivia are more depleted than the regions within Colombia (<−210‰).

Isoscapes are a useful tool in visualizing one specific signal. However, the generated maps offered little assistance in the classification of illicit cocaine samples to a specific sub-region within Bolivia, Colombia, or Peru. Correlation plots utilized in the original study^11^ were unsuccessful in elucidating any clear divisions amongst the data. These complications arose not only due to the large variation exhibited in the data set as a whole but also within the sub-regions, i.e., Norte de Santander and Caquetá. Although direct interpretations from the isoscapes and analytical data were not possible, it was apparent that the independent information provided by the analytical measurements could provide the leverage to assess the characteristics of any one sample. Therefore, additional exploratory and chemometrics methods were investigated to properly characterize clustering and perform classification tasks for the large volume of data obtained.

### Development of predictive framework

For decades, ‘-omics’ communities have applied multivariate methods to illuminate biogeochemical relationships (i.e., organic molecular, trace element abundances, and/or isotope amount ratios) between samples sharing similar geographic provenance. While geostatistical modeling may provide pathways for the regional classification of samples, there are also many powerful chemometric and machine learning approaches that are effective in the absence of geographic coordinates when regional labels are known. Support vector machine classification (SVM-C) and classification by projection onto latent structures using partial least squares (PLS-C), more commonly known as PLS-DA, are two highly used approaches in chemometrics. Unfortunately, the “black-box” approach of SVM-C hinders the follow-on interpretation of the machine output which might indicate an underlying process(es) responsible for the relationships between samples and their association with their regional label. Additionally, the selection of tuning parameters governing the complexity of the functions used to fit a SVM-C solution often result in an optimal solution that should be rigorously examined by validation testing. If used naively, highly favorable outcomes could seem very impressive at first glance but often are the result of over training, which translates to the model having specific expertise about the data under study but little common sense concerning what it hasn’t seen^28^.

As most data analysts are keenly aware, real world data are generally messy and most are virtually never “normal”. As a result, using traditional and rigorous statistical methods for assessing the data is not usually the best way to begin the analysis. This investigation began with an intensive exploratory data analysis (EDA) phase to gather knowledge about the properties of the data as well the opportunity to identify and remediate potential problems such as missing values, unequal measurement scales from fusing disparate data types, testing for multivariate normality, and identifying outliers. While not used exclusively, principal component analysis (PCA) and hierarchical agglomerative clustering (HCA) based upon Mahalanobis distances were used to rapidly assess the data for the presence of outliers and to assess the impact of various data transformations on the general formation of clusters. Most geochemical data is characterized as left-censored, which translates into missing data for samples for which the intended chemical measurement falls below the limit of detection (<LOD) and leads to much research into remediating the missing value(s). It is also routinely common to encounter highly skewed distributions which typically require a transformation of the affected variable to meet a fundamental assumption of multivariate normality for most follow on statistical methods. Samples which consistently displayed high residuals, high within class Mahalanobis distances for the multivariate case^29^, or were identified as suspect using Tukey’s method for the univariate case were tabulated and reviewed for consistency with expected results and trends in the cocaine data^30^. This approach led to the review of approximately 7% of the overall dataset, and samples that were considered mild or moderate outliers were returned to the data table.

Further results from routine EDA methods provided additional insight. For example, HCA and model based clustering (MBC) demonstrated that the majority of samples from the Cusco (N = 43) and Valle de Cauca (N = 20) regions clearly associated with the UHV samples *vs.* any other grouping. This observation is in agreement with the previously discussed trends known for δ^15^N values throughout the study area. Temporarily combining these regions into a supercluster while interrogating the rest of the data was supported by inspecting the Bayes Information Criterion (BIC) scores from model based clustering. MBC analysis also suggested the optimal number of clusters is much less than the number of classes which were defined for this investigation. However, additional EDA on the UHV-Cusco-Valle de Cauca supercluster indicated that when isolated, sub-clustering of the constituent regions as well as sub-regional classification was reasonably attainable as well. Further iterations of this type of analyses permitted the remaining data to be assigned to one of six identified superclusters.

Based upon these findings, an approach to divide the problem into a hierarchical series of binomial predictions based upon nested multivariate models was adopted. Hierarchical models which employ multiple classifiers is often better suited to making use of between-class relationships discovered in the data as compared to traditional “flat” modeling methods. Additionally, when the number of predictors is much less than the number of classes one wishes to distinguish, some compromise is likely to occur such as the recycling of predictors using recursive partitioning or interval based methods. The tree-structured hierarchical modeling approach (TSHC) adopted follows a similar strategy recently utilized for the hierarchical classification of watersheds by chemical signatures^31^. The methodology in this study departs from that strategy in the terminal step where instead of a regression step to predict geographical coordinates for the sample, a class label is assigned^32^. Additionally, while very potentially useful, the approach followed does not currently implement variable selection. The TSHC approach embeds the set of superclusters along with their sub-clusters into a hierarchical tree structure, using each nonterminal node as the host site for a unique classification model and each terminal node serving to assign the unique identifier (class label). When the TSHC model is given a sample represented by its alkaloid and isotope measurements, the predicted regional label of the sample is gradually restricted as it is passed through the series of classification steps.

**Figure 4:**
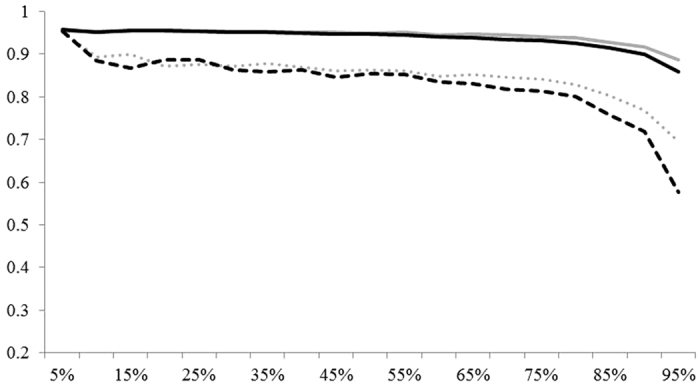
Mean accuracy of DKOPLS-C as function of sampling fraction hold out (grey solid), mean accuracy of SVM-C (black solid), mean MCC of DKOPLS-C (grey dotted), and mean MCC of SVM-C (black dashed).

While similar K-OPLS methods are described in literature^33^, it is believed that this is the first investigation which uses the hyphenation of discretization, projection, and discriminant methods to maximally separate multiple highly-overlapping classes in hierarchical fashion. The generalized approach to the prototype framework begins with preprocessing the data at the first node using discretization^34^. Applying discretization at this step in the analysis of the data served as a universal remediation strategy for missing data and moderating some outlier effects on data clustering. A Gaussian kernel was used to project the data into a higher dimension which was followed by PLS-C. Additionally, classification by SVM-C was also performed simultaneously to compare performance figures of merit later on. PLS_C Model refinement was performed via orthogonalization which removes latent orthogonal variation which often results in better predictions^35^. To ensure the overall framework was robust, an assessment of the framework’s predictive capability was studied using a Monte Carlo approach where the fraction of samples chosen during the stratified sampling step was varied in a stepwise manner. Figure 4 shows the accuracy of both DKOPLS-C and SVM-C determined by performing 100 iterations per 0.05 step and computing overall mean misclassification rate along with the Matthew’s correlation coefficient (MCC) among the nodes^36^. Additional hold-out studies show when an unknown illicit cocaine sample is compared to the database of geographically referenced samples, the model accurately classifies the unknown as belonging to one of the 19 sub-regional growing regions with an accuracy of approximately 96%.

### Analysis of illicit cocaine samples

The aim of the present investigation was to not only fully characterize the cocaine produced throughout South America but to also assess the origin of the cocaine that is being trafficked to the United States for illicit consumption. This laboratory currently analyzes more than 2,500 samples from domestic seizures each year. In recent years, the bulk of cocaine seizures have occurred in international waters. In 2014, approximately 43 metric tons of cocaine were seized in the eastern Pacific (EPAC) and Caribbean. This amount represents 79% of the total amount of bulk cocaine (seizures larger than 10 kg) seized by United States authorities and submitted to the laboratory in 2014.

**Figure 5:**
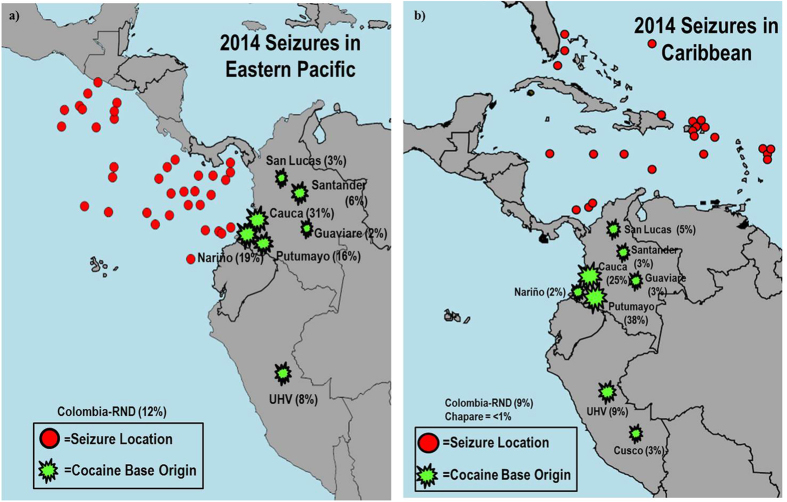
Maps illustrating 2014 cocaine seizures in (**a**) the eastern Pacific and (**b**) Caribbean with their respective sub-regional classifications. Both maps were created with SmartDraw software.

Figure 5 illustrates the seizure locations as well as the sub-regional origin classifications of samples analyzed from seizures in the EPAC and Caribbean. Classifications representing less than 1% of all samples analyzed are not shown. The majority of the cocaine seized in the EPAC originates from southwestern Colombia, with Cauca being the most dominant region (Fig. 5a). Nariño and Putumayo accounted for 19% and 16% of seized samples, respectively. More than 10% of the samples were classified as Colombia-Region Not Determined (Colombia-RND). This classification is given to samples which do not meet an adequate confidence level for making a final sub-regional determination, but all data overwhelmingly indicate the sample is of Colombian origin. Less than 10% of samples analyzed originated from Guaviare, San Lucas, Santander, and the UHV (Peru).

Caribbean-seized cocaine has significantly different origins versus the EPAC (Fig. 5b). Southwestern Colombia is by far the most dominant producer; however, most samples originated in Putumayo (38%). Cauca represents 25% of seized samples, similar to that of EPAC-seized cocaine. Nariño only accounts for 2% of samples, which is significantly less in comparison to the EPAC classifications. Again, San Lucas, Santander, and Guaviare each represent less than 10% of all Caribbean-seized cocaine. Seven percent of samples were assigned the Col-RND classification. One significant difference to note is with samples of Peruvian origin. There is a slightly higher occurrence of cocaine from the UHV (9%), but there are also samples originating from Cusco present (3%) in the Caribbean-seized samples. Interestingly, there are no samples originating from the Cusco region present in any of the cocaine seized in the EPAC.

While most samples analyzed in this laboratory are seized in the open ocean, cocaine seizures do occur within the continental United States (CONUS). For CY 2014, samples from seizures representing approximately five metric tons of cocaine have been analyzed. The majority of CONUS seizures occur in California, Texas, and Florida. Table 2 lists the predominant sub-regional classifications in each of these states. Again, southwestern Colombia is the most significant producer of cocaine seized within the United States with Cauca, Putumayo and Nariño representing 25%, 21%, and 13% of all CONUS seizures, respectively. Significant differences are observed upon comparison of the California and Texas seizures with those from Florida. Specifically, samples originating from Cauca and Putumayo are observed more on the east coast versus the western United States. However, there is significantly less cocaine originating from Nariño seized in Florida. This trend mirrors the classifications discussed from EPAC- and Caribbean-seized cocaine. Furthermore, cocaine originating from the regions of Antioquia and San Lucas were only observed in California and Texas. As observed for seizures in international waters, the destination for Peruvian cocaine differs from coast to coast. Peruvian cocaine originating from the UHV is mostly found in California and Texas, while only 2% of samples analyzed from Florida seizures were determined to be from the UHV. However, less than 1% of the cocaine seized in California and Texas is of Cusco origin.

In addition to the domestic samples analyzed each year, DEA foreign offices also submit cocaine seizures for geo-sourcing purposes (approximately 500/year). Foreign seizures are important for capturing authentic cocaine data before it is trafficked and for highlighting the movement of cocaine to other regions of the world. With the presented methodology, it is also possible to discover new, emerging coca growing regions within South America. Recently, this laboratory received 25 cocaine samples from a large “aircraft drop” in Uruguay. After collecting alkaloid and stable isotope data, preliminary evaluation indicated the samples may have originated from Bolivia. However, upon further inspection by the data analysis scheme, the samples appeared to be unlike any other sample within our database. The isotope data, in particular, was very different from any authentic Bolivian samples. The δ^13^C data was depleted (−35.6‰) suggesting the coca grew in a region of lower altitude. Nitrogen isotope data was more enriched (−10.3‰) than expected from samples in the Chapare Valley. Based on the gradual decrease in δ^15^N incorporation upon moving further south of the equator, it was determined these samples likely originated north of the Chapare Valley. Deuterium and δ^18^O values suggested a wetter environment in comparison to the Chapare Valley due to enrichment (−181.9 and 19.6‰). All of the isotope data indicated the cocaine was processed from coca leaf originating north of the Chapare Valley, the only Bolivian region of which we had prior knowledge. Further information obtained during the course of the investigation validated our conclusion. Intelligence reports from the pilot responsible for carrying the cocaine stated the plane did in fact depart from Beni, Bolivia. Prior to this seizure, there had been no evidence of coca cultivation in this area, which lies north of the Chapare Valley.

## Discussion

The ability to provide regional classifications has strengthened cocaine sourcing capabilities by providing a more detailed analysis of the cocaine being trafficked out of South America. In general, most of the cocaine trafficked out of Colombia is produced with coca grown in the southwestern regions of Cauca, Putumayo, and Nariño. Additionally, samples originating from Santander, Guaviare, and San Lucas are often detected in bulk seizures. This data coincides with the decreased eradication efforts in southwestern Colombia due to the presence of terrorist organizations.

In addition to current cultivation and production trends, the movement of cocaine for trafficking is also observed from the combination of seizure location and sub-regional classifications. Differences between cocaine seized in the EPAC versus that in the Caribbean suggest various trafficking routes and/or multiple drug trafficking organizations utilizing these routes. The same observation holds within the continental United States when comparing seizures in California and Texas to those made in Florida.

Comparing unknown samples to a database of authentic geographically referenced cocaine samples allows for the discovery of new growing regions within South America. The extensive statistical treatment of the data is instrumental to this process as it provides dynamic output that can easily be interpreted by a user. Combining the output with our knowledge of all analytical trends observed for cocaine, we are confident in assessing the true location of any potential new cultivation areas even in the absence of referenced coca leaf.

In summary, origin classification of cocaine to one of 19 known coca-growing regions in Colombia, Peru, or Bolivia is now possible. Routine laboratory analyses consisting of alkaloid and stable isotope determinations, combined with complex statistical analyses, provide a reliable tool by which cocaine movement, cultivation, and interdiction efforts may be assessed. The dynamic nature of the statistical analysis allows for the addition of new growing regions, and thus is capable of further improving the current scientific intelligence product. The presented investigation is the product of the exemplary coordination of traditional analytical chemistry and isotope biogeochemistry and has far reaching potential outside forensic science.

## Methods

### Materials

All chemicals and solvents used were reagent grade or better and were obtained from Sigma-Aldrich. All internal standards were prepared as described in preceding studies^37^,^38^.

### Authentic coca leaf

Coca leaf samples (N = 572) were collected from 19 known growing regions throughout Bolivia (N = 58), Colombia (N = 361), and Peru (N = 153). The latitude and longitude of each sample were recorded upon harvest. Samples were thoroughly dried and stored in paper bags within a temperature- and humidity-controlled vault. Prior to analysis, the coca leaf was ground to a fine powder with a Wiley mill.

### Isolation of cocaine from bulk coca leaf

Although this process was completed in a controlled laboratory setting, the chemical extraction is identical to that utilized during clandestine cocaine processing. The isolation of cocaine and trace alkaloids was accomplished using modified trap column methodology originally utilized by Moore *et al*.^4^ Approximately 40 g of ground, dry coca leaf was treated with approximately 40 mL of saturated aqueous sodium bicarbonate and mixed well. The basified leaf was then extracted with 1 L of water-saturated toluene for 3 hours. The concentrated extract was then filtered and passed through a column packed with a mixture of 25 g Celite and 12.5 mL of 0.36 N sulfuric acid. The column was washed with 75 mL of water-saturated toluene followed by 75 mL of water-saturated chloroform. All eluates were discarded. Cocaine and related alkaloids were liberated from the column with 60 mL of water-saturated chloroform containing 250 μL diethylamine. The eluate was evaporated in *vacuo* to an oil. The oil was reconstituted in 25 mL of dichloromethane and utilized for all remaining analyses.

### Alkaloid analyses

Each sample was prepared for quantitation by diluting 100 μL of dichloromethane solution with 1 mL of 0.9 mg/mL isopropylcocaine in chloroform. Samples were quantitated via gas chromatography with flame ionization detection (GC/FID) methodology as originally described by Piñero *et al*.^37^.

Approximately 500 μL (or 4 mg equivalents of cocaine) of the cocaine solution were dried and prepared for analysis as previously described for trace alkaloid analysis^38^. Relative amounts of tropacocaine and trimethoxycocaine were determined via GC/FID after derivatization with N-methyl-N-(trimethylsilyl)-trifluoracetamide (MSTFA).

Approximately 150 μL (or 1 mg equivalents of cocaine) of the cocaine solution were dried and prepared for truxilline analyses. The relative amount of total truxillines present in all samples was determined via GC/ECD after an extraction and derivatization procedure that has been previously reported by Moore *et al*.^39^.

Initial quantitative analyses were completed with an Agilent (Palo Alto, CA) 7890A gas chromatograph fitted with a 30 m × 0.25 mm ID fused-silica capillary column coated with 0.25 μm DB-1 (Agilent). The instrument was operated in split mode (25:1). All trace alkaloid determinations were performed with an Agilent 7890A gas chromatograph fitted with a 30 m × 0.25 mm ID fused-silica capillary column coated with 0.25 μm DB-1701 (Agilent). Truxilline analyses were completed in splitless mode, while tropacocaine and trimethoxycocaine were determined with split mode (50:1).

### Basic alumina column chromatography

Approximately 8 mL (or 50 mg equivalents of cocaine) of the cocaine solution were dried down in a centrifuge tube. The remaining oil was reconstituted in approximately 1 mL of chloroform. A chromatographic column was prepared with 10.0 g of basic aluminum oxide containing 4.0% water. The chloroform solution was quantitatively transferred to the column. Approximately 30 mL of chloroform was then added to the column. The first 10 mL of eluate were collected in a centrifuge tube. The collected chloroform was dried under nitrogen and then quantitatively transferred to a 4 mL vial with ether. The solution was dried to a powder by heating at 75 °C. The resulting powder was accurately weighed in preparation for isotopic analyses.

### Isotope Ratio Mass Spectometry (IRMS)

Carbon and nitrogen isotope ratio analyses were determined using an elemental analyzer (EA)(Costech Analytical Technologies Inc., Valencia, CA) interfaced with a Delta Plus XP isotope ratio mass spectrometer (Thermo Fisher Scientific Inc., Bremen, Germany). Typically, 0.9–1.2 mg of cocaine was weighed into a tin capsule (Costech Analytical Technologies Inc.) and introduced into the EA using a Costech Zero-Blank autosampler (Costech Analytical Technologies Inc.). The EA reactor tubes were comprised of two quartz glass tubes filled with chromium(III) oxide/silvered cobaltous oxide and reduced copper, held at 1040 °C and 640 °C for combustion and reduction, respectively. A trap filled with magnesium perchlorate was used to remove water from generated combustion gases, and a post-reactor GC column was maintained at 65 °C for separation of evolved N_2_ and CO_2_. Helium (99.999% purity, ARC3 Gasses, Richmond, VA) was used as the carrier gas, and the system head pressure was adjusted to achieve a measured flow of 90 mL/min. Data was acquired and processed using ISODAT 3.0 software (Thermo Fisher Scientific Inc., Bremen, Germany).

Sample sequences were bracketed by an internally calibrated atropine secondary standard (TCI, St. Louis, Missouri), typically at intervals of one standard every seven samples. The atropine secondary standard was calibrated to primary isotopic standard materials relative to Vienna Pee Dee Belemnite (VPDB) for carbon and AIR for nitrogen. System reproducibility was consistently 0.1‰ and 0.2‰ or better for all EA-IRMS δ^13^C and δ^15^N measurements, respectively.

Hydrogen and oxygen isotope ratio analyses were determined using a thermo-chemical elemental analyzer (TCEA) interfaced with a Delta V isotope ratio mass spectrometer (Thermo Fisher Scientific Inc.). In TCEA-IRMS analysis, approximately 0.20 to 0.25 mg of purified cocaine was accurately weighed into silver capsules (Costech Analytical Technologies Inc.) and introduced into the TCEA using a Costech Zero-Blank isolated autosampler (Costech Analytical Technologies Inc.). The capsules were pyrolyzed in the TCEA-IRMS system into H_2_, CO, and C by passing through a ceramic reactor filled with a glassy carbon tube and glassy carbon pieces at 1400 °C. A post TCEA reactor GC column was maintained at 70 °C for separation of evolved H_2_ and CO. Helium (99.999% purity) was used as the carrier gas, and the system head pressure was adjusted to achieve a measured flow of 90 mL/min. Data was acquired and processed using ISODAT 3.0 software.

Sample sequences were bracketed by an internally calibrated C-28, C-34 and atropine secondary standards (TCI, St. Louis, MO) as well as benzoic acid primary standard (IAEA-601), typically at intervals of one standard every seven samples. The secondary standards were calibrated to primary isotopic standard materials relative to Vienna Standard Mean Ocean Water (VSMOW) for hydrogen and AIR for oxygen. System reproducibility was consistently 3.0‰ and 0.4‰ or better for all TCEA-IRMS δ^2^H and δ^18^O measurements, respectively.

### Data Landscapes

All data landscapes were generated within the ArcGIS Platform (Esri, Redlands, CA) with the ordinary kriging interpolation method. The ArcGIS methods utilized to create the predicted landscapes were employed to yield optimal Root Mean Square (RMS) error and Average standard RMS error results with the least amount of artifacts. Whenever possible, care was taken to use similar parameters for each surface. Ordinary kriging with 1^st^ order trend removal was used for trimethoxycocaine, total truxillines, tropacocaine, and δ^2^H. Ordinary kriging with no trend removal was used for δ^13^C and δ^15^N. All isoscapes had the “neighbors to include” parameter changed to values between 14 and 16 and the “stations to include” parameter changed to values between 12 and 14. Default ArcGIS values were used for all other parameters. Each measurement is displayed only in terms of the geographical coordinates associated with the authentic samples.

### Framework Development

Customized code utilized for the prototype framework was developed in house with Mathworks MATLAB^©^ rev. 2014a; however, the following commercial software and/or publically available code have been evaluated to achieve similar results if assembled: MATLAB and Statistics Toolbox Release 2014a (The MathWorks, Inc., Natick, Massachusetts, United States), PLS_toolbox^©^ Ver 8.0 (Eigenvector Research Inc. Wenatchee, WA 98801, www.Eigenvector.com), and CAIM^©^ 2009 for MATLAB by Guangdi Li (available through the Mathworks Users Code Exchange). A great deal of EDA was completed prior to developing the final methodology and framework. While not used exclusively, the EDA_Toolbox V2.0 for MATLAB^©^ provides a convenient suite of functions suitable for exploring data using both supervised and unsupervised methods^40^.

## Additional Information

**How to cite this article**: Mallette, J. R. *et al*. Geographically Sourcing Cocaine’s Origin – Delineation of the Nineteen Major Coca Growing Regions in South America. *Sci. Rep.* **6**, 23520; doi: 10.1038/srep23520 (2016).

## Figures and Tables

**Table 1:** Average analytical responses of authentic cocaine samples from 19 South American coca-growing regions.

Growing Region (N = )	Tropacocaine	Trimethoxycocaine	Truxillines	δ^15^N	δ^13^C	δ^2^H	δ^18^O
Amazonas (19)	0.41 (0.17)	0.69 (0.20)	3.9 (0.6)	−4.3 (0.9)	−35.7 (0.5)	−210.9 (10.0)	12.9 (1.9)
Antioquia (20)	0.61 (0.31)	0.26 (0.09)	6.2 (1.3)	−7.6 (1.5)	−34.3 (0.4)	−228.7 (4.7)	10.4 (1.1)
Arauca (20)	4.86 (2.72)	0.01 (0.00)	24.1 (9.7)	−7.4 (2.2)	−33.4 (0.5)	−167.8 (7.0)	23.8 (2.1)
Caquetá (30)	0.48 (0.56)	0.30 (0.34)	9.7 (5.1)	−0.9 (2.0)	−34.3 (0.4)	−189.0 (17.8)	18.6 (3.1)
Cauca (12)	1.28 (2.01)	0.24 (0.19)	7.4 (5.0)	−6.0 (2.3)	−33.3 (1.3)	−199.0 (9.0)	16.3 (1.8)
Chapare (56)	0.26 (0.06)	0.16 (0.03)	2.7 (0.3)	−12.3 (1.2)	−34.5 (0.3)	−220.1 (15.2)	15.1 (3.0)
Chocó (20)	0.75 (0.45)	0.50 (0.14)	3.5 (0.8)	−8.8 (0.6)	−34.9 (0.5)	−218.5 (3.9)	11.3 (2.5)
Cusco (41)	0.12 (0.04)	0.27 (0.06)	4.1 (1.6)	−9.5 (0.8)	−33.7 (0.4)	−226.7 (14.1)	18.3 (3.4)
Guaviare (18)	0.14 (0.11)	0.59 (0.19)	4.3 (1.5)	−5.2 (1.4)	−34.9 (0.6)	−191.9 (7.6)	19.2 (2.1)
Meta (19)	0.73 (0.49)	0.73 (0.24)	5.0 (1.4)	−5.5 (1.7)	−34.8 (0.4)	−223.6 (7.1)	12.0 (1.8)
Nariño (33)	0.53 (0.41)	0.47 (0.30)	4.2 (3.3)	−8.8 (1.7)	−35.5 (0.8)	−199.1 (5.1)	16.1 (1.8)
Norte de Santander (15)	3.52 (4.39)	0.35 (0.37)	26.4 (24.7)	−4.0 (1.8)	−34.4 (1.0)	−207.5 (4.6)	14.4 (1.1)
Putumayo (36)	0.75 (1.19)	0.60 (0.21)	7.6 (3.4)	−4.5 (2.0)	−34.4 (0.7)	−205.6 (9.0)	18.7 (2.1)
San Lucas (20)	2.40 (1.75)	0.04 (0.11)	22.2 (5.4)	−4.8 (2.3)	−33.7 (0.6)	−181.2 (7.1)	19.5 (1.6)
Santander (18)	0.51 (0.30)	0.62 (0.39)	4.7 (2.7)	−9.1 (1.3)	−35.2 (0.6)	−197.2 (9.8)	15.5 (1.5)
UHV (96)	0.17 (0.06)	0.19 (0.04)	3.7 (0.6)	−8.1 (1.1)	−35.0 (0.4)	−219.0 (11.1)	15.1 (2.6)
Valle de Cauca (19)	1.00 (0.45)	0.19 (0.04)	4.1 (0.7)	−10.1 (1.4)	−34.7 (0.4)	−225.0 (2.4)	12.2 (0.9)
Vaupés (18)	0.56 (0.32)	0.50 (0.18)	4.7 (1.1)	−6.8 (1.9)	−35.7 (0.6)	−194.2 (5.7)	17.5 (1.4)
Vichada (19)	1.06 (0.07)	1.77 (0.45)	6.7 (0.4)	−4.7 (0.2)	−35.6 (0.1)	−209.1 (5.0)	13.4 (0.3)

Alkaloid values are represented as % relative to cocaine. Isotope ratios are represented in ‰. The standard deviation for each measurement is shown in parentheses.

**Table 2:** Percent of total samples per regional classification for seizures within the continental United States (CONUS) and select states.

Classified Region	Percent of All CONUS Samples			
	All CONUS^1^	CA	TX	FL
Antioquia	4	4	5	–
Cauca	25	25	21	37
Guaviare	2	2	3	2
Nariño	13	11	15	3
Putumayo	21	15	22	37
San Lucas	3	4	3	–
Santander	4	3	5	3
UHV	8	11	8	2
Cusco	<1	<1	<1	2
Col-RND	14	15	12	14

^1^Total samples: CONUS = 590; CA = 186; TX = 232; FL = 57.
